# Retention in Differentiated Care: Multiple Measures Analysis for a Decentralized HIV Care and Treatment Program in North Central Nigeria

**DOI:** 10.4172/2155-6113.1000756

**Published:** 2018-02-13

**Authors:** Patricia A Agaba, Becky L Genberg, Atiene S Sagay, Oche O Agbaji, Seema T Meloni, Nancin Y Dadem, Grace O Kolawole, Prosper Okonkwo, Phyllis J Kanki, Norma C Ware

**Affiliations:** 1 Faculty of Medical Sciences, University of Jos, Nigeria; 2 Johns Hopkins University Bloomberg School of Public Health, Baltimore, MD, USA; 3 Harvard TH Chan School of Public Health, Boston, MA USA; 4 APIN Centre, Jos University Teaching Hospital, Jos, Nigeria; 5 AIDS Prevention Initiative Nigeria Lte, Abuja, Nigeria; 6 Harvard Medical School, Boston MA, USA; 7 Brigham & Women’s Hospital, Boston, MA, USA

**Keywords:** Differentiated HIV Care, Africa, Antiretroviral therapy, HIV treatment, Retention, Decentralization, measure, Nigeria

## Abstract

**Objective:**

Differentiated care refers collectively to flexible service models designed to meet the differing needs of HIV-infected persons in resource-scarce settings. Decentralization is one such service model. Retention is a key indicator for monitoring the success of HIV treatment and care programs. We used multiple measures to compare retention in a cohort of patients receiving HIV care at “hub” (central) and “spoke” (decentralized) sites in a large public HIV treatment program in north central Nigeria.

**Methods:**

This retrospective cohort study utilized longitudinal program data representing central and decentralized levels of care in the Plateau State Decentralization Initiative, north central Nigeria. We examined retention with patient- level (retention at fixed times, loss-to-follow-up [LTFU]) and visit-level (gaps-in-care, visit constancy) measures. Regression models with generalized estimating equations (GEE) were used to estimate the effect of decentralization on visit-level measures. Patient-level measures were examined using survival methods with Cox regression models, controlling for baseline variables.

**Results:**

Of 15,650 patients, 43% were enrolled at the hub. Median time in care was 3.1 years. Hub patients were less likely to be LTFU (adjusted hazard ratio (AHR)=0.91, 95% CI: 0.85-0.97), compared to spoke patients. Visit constancy was lower at the hub (−4.5%, 95% CI: −3.5, −5.5), where gaps in care were also more likely to occur (adjusted odds ratio=1.95, 95% CI: 1.83-2.08).

**Conclusion:**

Decentralized sites demonstrated better retention outcomes using visit-level measures, while the hub achieved better retention outcomes using patient-level measures. Retention estimates produced by incorporating multiple measures showed substantial variation, confirming the influence of measurement strategies on the results of retention research. Future studies of retention in HIV care in sub-Saharan Africa will be well-served by including multiple measures.

## Introduction

“Test-and-treat” approaches to HIV services in resource-scarce settings require new, more flexible models of care capable of meeting the varying needs of increasing numbers of patients. Service models designed to address these differing needs in a client-centered framework have come to be known collectively as “differentiated care” [1,2]. Increasing emphasis on differentiated care has brought with it a shift in service settings - from large, hospital-based HIV-specialty clinics to communities.

One such shift has been known as “decentralization.” Decentralization of HIV care means relocating services from centralized sites to peripheral centres, which are geographically closer to patients. Decentralization was introduced in 2002 by the World Health Organization as a strategy for expanding access to antiretroviral therapy (ART) [3]. Besides improving access, decentralization also aims to improve health outcomes and retention in care [4].

Retention is a crucial indicator for monitoring and evaluating HIV care and treatment programs, particularly in the test-and-treat era, when patients may begin ART before experiencing symptoms. High retention levels have been associated with improved ART adherence, slower disease progression, improved survival and reduced infectiousness [5-7]. Despite its importance, there is no recognized “gold standard” measure of retention [8-12]. Most published studies in low and middle-income countries, especially in sub-Saharan Africa, have relied on cohort or cumulative measures [13-17]. Cohort rates report retention at the end of a specific calendar period among a cohort of patients entering care and followed over time. Cumulative retention counts patients “ever initiated on ART” or “currently on ART.” These summary measures do not capture the variability that occurs in retention over time, impacting service utilization and resulting clinical outcomes. Alternative measures of retention are available [11,12,18-20] and could be used to improve research on retention in HIV treatment and care in SSA.

Our study addresses this gap, employing multiple measures to explore patterns of retention in a decentralized HIV care program in Nigeria. Our objective was to compare patient retention in central versus decentralized treatment sites.

## Materials and Methods

### Study setting

To improve access to HIV care in Plateau State, north central Nigeria, the HIV treatment program at Jos University Teaching hospital (JUTH), in the state capital of Jos, launched the Plateau State Decentralization Initiative in 2007. A “hub-and-spoke” model of decentralization was used in which the JUTH-HIV specialty clinic (“hub”) was linked to 13 community hospitals in surrounding semi-urban and rural areas (“spokes”). Community hospitals were empowered to initiate and maintain patients on ART close to their residences [21]. Community hospital clinics were linked to 47 primary health care clinics (PHCs) providing HIV education, prevention and referral services.

ART eligibility in the program was determined by clinical staging and CD4+ cell count level, following national guidelines at the time [22,23]. Eligible individuals initiated first-line ART (1-NNRTI and 2-NRTIs), while individuals not meeting eligibility criteria were enrolled for pre-ART care. Dispensing of ART occurred monthly, at scheduled clinic visits. Monitoring of ART efficacy and toxicity was performed on schedule using standard clinical procedures and laboratory tests, including CD4+ cell counts and HIV plasma viral load (VL) assays.

### Study sample

Persons included in this study were HIV-1 infected, treatment-naïve patients (aged ≥ 15 years) at the time of enrolment in care. We followed patients between January 2008 (study baseline) and December 2012 (study follow-up end point). Those included were enrolled in the JUTH HIV specialty clinic (the “hub”) or at one of the 13 community hospital “spoke” treatment sites. Only patients enrolled in care for at least six months at the study end point were included, in order to fully examine longitudinal retention. Data were extracted from electronic medical records [24] and included baseline information on demographic characteristics (i.e., age, sex, education level, occupation, marital status), baseline clinical status (e.g. WHO clinical stage), laboratory measures (e.g. HIV VL and CD4+ cell count), ART initiation dates and follow-up clinical visit dates.

Upon entry into the care and treatment program and following informed consent, all patients were assessed for ART eligibility according to current Nigerian national treatment guidelines [22,23]. All ART- eligible patients were placed on ART following a clinical examination and baseline laboratory tests, which included hematology, clinical chemistries, CD4+ cell count and VL enumeration. Patients were given an initial 30 day supply of ART. Following the first prescription pick-up, refills were obtained on a monthly basis. Laboratory tests were repeated every six months unless an earlier evaluation was medically necessary. All patient data were maintained in electronic databases.

For the analyses reported here, we included patients who were newly enrolled in HIV care between January 2008 and June 2012, to ensure at least 6 months of follow-up time for the evaluation before data censure (December 2012). All patients were at least 15 years of age at enrolment. Patients who had ART experience prior to enrolling in the program were excluded.

### Retention measures

We employed a multiple outcome measurement strategy to assess retention in the hub-and-spoke network. Patient-level and visit-level measures were included to ensure comparability with other studies. Patient-level assessment consisted of two summary measures. The first summary measure - *retention at fixed-time points* - reports the proportion of the study population with a clinic visit within 180 days of three fixed time points: 12, 24 and 36 months after enrolment. The second summary measure - *loss to follow-up (LTFU)* - cumulatively estimates the proportion of the population: (a) without a clinic visit for more than 180 days at the end of the follow-up period; or (b) with only one visit during the follow-up period.

We also used two visit-level longitudinal measures to assess patterns of retention over time. A *gap in care* is a period of >180 days between clinic visits, with the patient having subsequent visits following the gap. *Visit constancy* is defined as six-month intervals (person-periods) for each patient from the time of enrolment through the study endpoint date. The number of visits within each interval was counted. *Visit constancy* estimates continuity in care by determining the proportion of six-month person-periods with at least one clinical visit.

### Ethics statement

This study was approved by the Institutional Review Board (IRB) at the Harvard T.H. Chan School of Public Health and the Committee on Human Studies at Harvard Medical School, Boston, MA. All consent forms were approved by the Harvard School of Public Health IRB, the National Health Research Ethics Committee (NHREC) of Nigeria, and the institutional review boards at the decentralized treatment sites. Only patients in the treatment program who provided written consent for care and for use of their stored data in future studies were included in this analysis.

### Statistical analysis

Descriptive statistics were used to assess baseline demographic and clinical characteristics of the study sample overall, and stratified by type of site (hub vs. spokes). Chi-square and Wilcoxon rank sum tests were used to examine differences in patient characteristics and retention outcomes by site type, including fixed time-point retention at 12, 24 and 36 months, LTFU, gaps in care and visit constancy. We examined the proportion of contiguous visits associated with a gap (>180 days since last visit), the median number of days since last visit among those with more than one clinical visit, and the gap duration for individuals with gaps in care. Finally, we examined the proportion of person-periods associated with at least one clinical visit.

Regression analysis was performed separately for each retention outcome. We first examined bivariate associations between the main exposure of interest (i.e., hub vs. spoke care site) and each outcome. Adjusted models included the main exposure of interest (site type) and all available demographic and clinical characteristics. A time-varying indicator was included in survival and longitudinal models to indicate pre or post-ART initiation time for each patient. A time-fixed indicator of any ART use was used in person-level models.

Logistic regression models with robust variance for clustering by site were used to examine retention at fixed time points (12, 24 and 36 months). Baseline time-fixed covariates were included in the model in addition to the main exposure (hub vs. spoke).

LTFU was examined using survival analysis methods. The Kaplan-Meier estimator was used to estimate time from enrolment in care to LTFU, comparing patients who enrolled at hub vs. spoke sites. Censoring was defined as having had at least one clinical visit within 180 days from the study end date. Cox regression analysis was used to examine the differences in LTFU for hub vs. spoke sites, adjusting for covariates. Survival analysis was restricted to those with >1 visit.

Visit constancy was examined using linear regression with generalizing estimating equations (GEE) for repeated measures among patients with exchangeable correlation structure. Visit constancy was time-updated at each six-month person-period and expressed as a percentage.

Gaps in care were examined using logistic regression with GEE with exchangeable correlation structure for repeated measures among patients. Gaps in care were examined only among patients with >1 visit.

Statistical significance was set at p<0.05. Stata statistical package version 13 (College Station, Texas, USA) was used for analyses.

## Results

### Characteristics of patients

A total of 16,816 patients were enrolled at hub and spoke sites during the study period. We excluded 1,166 patients because they had fewer than 180 days in care at the time of data censoring on December 31, 2012. The final sample included 15,650 patients with 128,017 total visits, of which 6,761 patients (43%) were enrolled at the hub.

Table 1 presents baseline demographic and clinical characteristics of the study sample. Median baseline CD4+ cell count for the entire sample at enrolment was 197 cells/mm^3^ (interquartile range (IQR): 100- 337). Sixty-six percent of the cohort was female. Median age at baseline was 33 years (IQR: 28-40) and median time since enrolment in care was 3.1 years (IQR: 1.9-4.1).

Comparisons across site type showed that a higher proportion of spoke versus hub patients were female (p<0.0001). They were also slightly younger (p<0.0001), more likely to be married (p<0.0001), more likely to have no formal education (p<0.0001) and less likely to have WHO stage III/IV illness at baseline (p<0.0001) than those from the hub site. There was no difference in median baseline CD4+ cell count between hub and spoke sites (p=0.45).

### Retention measures

Results of analyses examining differences in retention by site type using the four retention measures included in this study appear in Table 2. Data from each of these measures are grouped into larger “patient- level” and “visit-level” categories and presented below.

### Patient-level measures

#### Retention at fixed time-points

Using the summary measure of retention, 65.5%, 53.6% and 46.4% of the sample respectively were alive and in care at 12, 24 and 36 months from the time of enrolment. Hub patients were more likely than spoke patients to be alive and retained in care at 12, 24 and 36 months following enrolment (p<0.0001, Table 2).

#### Loss to follow-up (LTFU)

**Figure 1:**
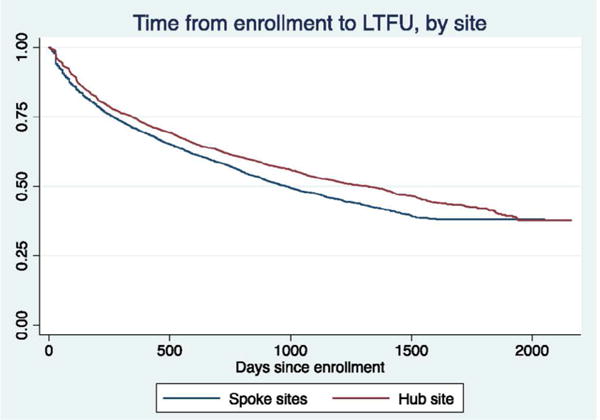
Kaplan-Meier plot showing the probability of being lost-to-follow-up (LTFU) from the time of enrolment to study endpoint date, by the hub vs. spoke sites.

Spoke sites had a higher proportion of patients who dropped out of care after only one visit, compared to the hub (22% vs. 19.4%, p<0.0001). The proportion of patients LTFU at the end of the observation period was 56.4% vs. 58.0% (p=0.05) for hub and spoke sites respectively, with hub patients having longer median time since loss at the study end date (1042 vs. 745 days, p<0.0001, Table 2). Survival methods, including the Kaplan-Meier estimator and the log rank test, confirmed shorter median time from enrolment in care to LTFU (2.7 years vs. 3.6 years), comparing spokes to the hub site (Figure 1).

### Visit-level measures

#### Gaps in care

Among patients with >1 visit, median time between visits was significantly longer for hub patients (69 days), than for patients at spoke sites (35 days) (p<0.0001; Table 2). There were more patients at the hub with at least one gap in care (43.4% vs. 28.4%, p<0.0001). A higher proportion of hub visits than spoke visits followed gaps in care (8.2% vs. 3.8%, p<0.0001). However, the median length of gaps was longer at spoke sites (224 days vs. 206 days, p<0.0001).

#### Visit constancy

Hub patients had a lower average proportion of patient-periods with at least one clinical encounter (70.2%), compared with spoke patients (71.7%, p<0.0001).

#### Adjusted models

Retention at 12, 24 or 36 months did not differ between hubs and spoke sites after adjusting for patient-level factors (data not shown). Females (compared to males) and those who had ever initiated ART (compared to those who had not) were more likely to be retained at 12, 24 and 36 months.

Table 3 presents results from models examining visit constancy by site, adjusted for socio-demographic and clinical characteristics, as well as year of enrolment in care. Enrolment at the hub, compared to spoke sites, was associated with 4.5% lower visit constancy over time (95% CI: −5.5, −3.5) after adjusting for patient factors.

Table 4 presents adjusted associations between type of site and gaps and LTFU. Adjusted logistic regression analysis showed that hub visits were almost twice as likely as spoke visits to follow a gap in care (adjusted odds ratio (AOR)=1.95, 95% CI: 1.83, 2.08). Adjusted Cox regression analysis showed enrolling in care at the hub vs. spoke sites was associated with decreased risk of being lost (adjusted hazard ratio (AHR)=0.91 (95% CI: 0.86, 0.97).

## Discussion

This large longitudinal cohort study evaluated patient retention in the Plateau State Decentralization Initiative, a hub-and-spoke network of HIV treatment and care in Plateau State, north central Nigeria. We used multiple patient- and visit-level measures to assess retention in the central “hub” and the decentralized “spoke” sites. Our measure of retention at fixed time points showed retention overall ranged from 65.5% at 12 months to 46.4% at 36 months of ART, with the hub performing better than the spokes at each time point. The hub also performed better on the loss to follow-up measure. Decentralized sites had better retention rates using the visit-level measures (gaps in care, visit constancy). Visit-level results suggest a more continuous pattern of visits and greater engagement in care among retained patients at the spokes, compared with the hub site. Additional research is warranted to understand the clinical significance of differences in visit constancy measures in this setting.

To our knowledge, this study is the first to use multiple outcome measures to evaluate patient retention in routine HIV care in sub- Saharan Africa. Visit-level measures, in particular, have not been used previously to evaluate retention in African HIV treatment programs. Results have shown that patient retention in this setting is comparable (using the gaps in care and visit constancy measures) to that obtained in public health care settings in North America, with site of care being a strong predictor of retention. For example, our results show fewer gaps in care at both central (43%) and decentralized (28%) sites, over a longer follow-up period, than reported in a study of retention among HIV-infected veterans receiving HIV care at US Veterans Health Administration (VHA) facilities (56%) [25]. Rates of retention represented as visit constancy were comparable at hub (70.2%) and spoke (71.7%) sites and the US VHA (73%), as reported in a separate study, again using a shorter follow-up period [12].

Previous studies comparing rates of retention in decentralized vs. more central facilities have consistently revealed better retention at decentralized care sites [7,26-29]. These studies have relied upon summary measures of retention at fixed time points. In contrast, our results showed that decentralized sites did not perform as well as the hub using summary measures. However, rates of retention were higher at the spoke sites when using visit-level measurement approaches. Summary measure differences were driven primarily by higher loss to follow-up rates, particularly following initial clinic visits, at spoke facilities. Loss to follow-up may have been overestimated at all sites in our analysis, due to inability to identify and remove from the study population patients who had died or self-transferred outside the hub- and-spoke network [30].

One reason retention “as continuity of visits” was better at decentralized sites may be because these treatment facilities were closer to patients’ residences. Clinic visits are easier to keep when transport distances are shorter, reducing the time and expense required to keep appointments [31,32]. In contrast, better retention “over the long-term” may have been observed at the hub because the hub site had more resources and could provide better quality care. However, patients may have found traveling long distances to remain in care became less feasible over time.

A useful framework for considering results from multiple measures lies in the medication adherence literature. Vrijens et al. [33] describe adherence to medications as consisting of three quantifiable phases: initiation (beginning therapy), implementation (how well patients take medication while continuing therapy) and discontinuation (stopping medication use). These concepts are analogous to the processes important to retention over time: linking to and engaging in HIV care (initiation), maintaining regular contact with a care provider (implementation), and dropping out of care/being LTFU (discontinuation). Applying this framework, we see that hub patients were less likely to discontinue care, while spoke patients had better implementation, or more frequent contact with care providers, over time.

Loss to follow-up rates were higher in this study than reported for either other HIV treatment settings in sub-Saharan Africa or a larger evaluation of HIV treatment programs in Nigeria [16,34-36]. However, researchers and program evaluators have applied disparate definitions of LTFU, which makes comparison across settings difficult. For example, a review of patient retention in antiretroviral therapy programs from 33 cohorts in sub-Saharan Africa incorporated eight definitions of LTFU [37]. Empirical data from 111 facilities in Africa, Asia, and Latin America were used to recommend adopting >180 days since the last clinic visit as a standard LTFU definition, the definition employed in this study [38]. Further standardization across measures of retention is needed.

We did not include patient medication refills as part of our definition of a visit in this analysis. Future research that includes medication refills, as well as an examination of the relationship between measures (e.g. the association between visit constancy and fixed-time retention) may be important to fully characterize retention in this setting. Finally, there may be some unmeasured confounding driving the differences in retention by hub and spoke sites reported here.

The retention estimates produced by incorporating multiple patient- and-visit level measures into our analysis showed substantial variation, clearly confirming the impact of measurement strategies on the results of retention research and yielding insights that would have remained hidden had we relied upon summary patient-level measures alone. The higher retention levels observed in spoke sites using visit- level measures suggest decentralization results in a more continuous pattern of clinic visits – an observation wholly consistent with the experience, for patients, of being able to access care closer to home.

Our study does not include any biological measures reflecting the impact of retention on patient clinical outcomes. While viral suppression is the ultimate goal for patients and programs, viral load data were not sufficiently complete to warrant their inclusion in this analysis. Viral suppression can be thought of as a proxy measure of both patient retention and medication adherence, since both are necessary conditions to achieve suppression. However viral load data are sparse in resource-constrained environments. Future research aimed at understanding patient retention over time should consider how to incorporate frequently missing data on viral suppression as a measure of patient retention in resource-limited settings.

A large sample size, use of routine program data, and a study setting reflecting an innovative decentralization model are among the strengths of this study. Several limitations must also be considered. First, there may have been misclassification due to under-ascertainment of outcomes for those who dropped out of care and were considered lost to follow-up, as described above. We did not incorporate mortality estimates or consider death as a competing event for our retention analyses. Insufficient patient-tracing systems at all sites and incomplete or unreliable mortality data likely resulted in overestimates of patients considered not retained using the retention at fixed time points and loss to follow-up measures. Undocumented self-transfers across treatment sites within the hub-and-spoke system may also have contributed to overestimation of true patient losses [39]. Finally, it should be noted that given the large patient volume, this study may have been overpowered to detect differences in visit-level measures.

## Conclusion

The impact of decentralization upon retention in HIV treatment and care is complex and varies depending on how retention is operationally defined and measured. The operational definitions and measures chosen can have far reaching effects on results, with each having its own value and utility. Future studies aimed at understanding retention in HIV care will be well served by including multiple measures as a means of strengthening validity and precision. In view of the UNAIDS global 90-90-90 targets for HIV prevention, multiple measurement strategies that include viral suppression will enable programs to more accurately evaluate this important outcome.

## Figures and Tables

**Table 1:** Baseline characteristics of 15,650 patients followed from January 2008-December 2012 in hub and spoke HIV care sites in north central, Nigeria.

Characteristics	Total N=15,650	Hub N=6,761 (43%)	Spoke N=8,889 (57%)	p-value

Female, n (%)	10281 (66)	63	68	<0.0001

Median age, in years (IQR)	33 (28-40)	34 (28-41)	32 (27-40)	<0.0001

Median time since enrollment, in years (IQR)	3.1 (1.9-4.1)	3.7 (2.4-4.5)	2.7 (1.6-3.5)	<0.0001

Marital status, n (%)				
Single	3235 (21)	25	18	<0.0001
Married	8953 (57)	54	60	
Divorced/Separated/Widowed	3428 (22)	21	23	

Educational level, n (%)				
None	2944 (20)	17	21	<0.0001
Primary	3991 (27)	22	30	
Secondary	4643 (31)	31	31	
Tertiary	3344 (22)	30	17	
Missing	726 (5)			

WHO Stage, n (%)				
I	4428 (28)	24	32	<0.0001
II	2316 (15)	13	16	
III	2259 (14)	21	9	
IV	641 (4)	5	4	
Missing	6004 (38)	37	39	

Median CD4 at baseline (IQR)	197 (100-337)	199 (98-346)	195 (102-328)	0.28

Initiated ART, n (%)	10775 (69)	64	73	<0.0001

IQR: Interquartile Range

**Table 2:** Summary of retention measures (retention at 12, 24 and 36 months, lost-to-follow-up (LTFU), gaps in care and visit constancy comparing hub and spoke sites.

	Hub site	Spoke sites	p-value

Total number of patients at baseline	6761	8889	

**Retention at Fixed Time Points**			

Retention at 12 months			
N enrolled ≥ 12 months from study end date	6345	7873	
% remaining in care* at 12 months from enrollment	67.6	63.8	<0.0001

Retention at 24 months			
N enrolled ≥ 24 months from study end date	5461	5904	
% remaining in care* at 24 months from enrollment	55.1	52.3	<0.0001

Retention at 36 months			
N enrolled ≥ 36 months from study end date	4459	3574	
% remaining in care* at 36 months from enrollment	47.6	44.9	<0.0001

**LTFU**			

Dropped out after 1 visit, n (%)	1311 (19.4)	1969 (22.2)	<0.0001
LTFU (≥ 180 days since any patient contact at study end)	3814 (56.4)	5154 (58.0)	0.05
Median time to LTFU, in years (IQR)	4.4 (3.4-4.9)	3.4 (2.3-4.3)	<0.0001
Median time since LTFU at end of study, in years (IQR)	2.9 (1.5-3.9)	2.0 (1.0-3.1)	<0.0001

**Gaps in Care**			

Patients with >1 visit	5450	6920	

Had at least 1 gap during follow-up, n (%)	2367 (43.4)	1962 (28.4)	<0.0001

Follow-up visits among those with >1 visit, n	45,103	67,264	

Median time between visits overall** (IQR)	69 (28-125)	35 (28-77)	<0.0001

Median length of longest time between visits, in days (IQR)	181 (147-223)	141 (91-196)	<0.0001

Gaps (> 180 days between visits), n (%)	3690 (8.2)	2558 (3.8)	<0.0001

Median length of gaps, in days (IQR)	206 (192-270)	224 (196-280)	<0.0001

Median length of longest gap, in days (IQR)	244 (200-315)	238 (203-322)	0.55

**Visit-Constancy**			

Total number of 6 month periods	47,362	49,290	

Median number of periods/patient (IQR)	8 (6-9)	6 (3-8)	<0.0001

Mean number of visits within period (SD)	1.1 (1.3)	1.5 (1.9)	<0.0001

Mean percentage of periods/patient with at least 1 visit (SD)	70.2 (34.4)	71.7 (33.8)	<0.0001

Percent of periods with time-updated constancy > 80%	55.7	57.0	<0.0001

* Remaining in care defined as having had an appointment within at least 180 days from year end

LTFU: Lost-to-Follow-Up; IQR: Interquartile Range; SD: Standard Deviation

** Visits include clinical visits and visits with only lab measures (CD4+ cell count and/or viral load assay) without a clinical visit

**Table 3:** Adjusted estimates of visit constancy, comparing the hub and spoke sites, expressed as percentage of 6 month person-periods with at least one clinical visit.

	Estimate (95% CI)

Constant	76.69
Hub vs. Spoke site	−4.49 (−3.53, 5.45)

Age (per 10 years)	0.06 (−0.02, 0.14)

Female (vs. Male)	3.58 (2.54, 4.61)

Disease status at baseline*	−8.08 (−9.18, −6.99)

Post-ART initiation (vs. pre-ART)	−3.19 (−3.72, −2.65)

Marital status	
Single	Reference
Married	4.29 (3.03, 5.56)
Other (widowed, separated, divorced)	4.24 (2.74, 5.75)

Education	
None	Reference
Primary	3.82 (2.41, 5.23)
Secondary/Tertiary	5.20 (3.81, 6.60)

* CD4<200 and/or WHO Stage 3-4 vs. CD4 > 200 and/or WHO Stage 1-2

**Table 4:** Odds ratios (OR) for gaps in care and hazard ratios (HR) of being lost-to-follow-up by patient characteristics.

	Gaps in care *n*=12370		LTFU *n*=15650	

	OR (95% CI)	Adjusted* OR (95% CI)	HR (95% CI)	Adjusted* HR (95% CI)

Hub (vs. Spoke)	2.18 (2.05-2.31)	1.95 (1.83-2.08)	0.84 (0.80-0.89)	0.91 (0.85-0.97)

Age (per 10 years)	1.00 (0.99-1.00)	1.00 (0.99-1.00)	0.99 (0.99-1.00)	0.98 (0.96-1.00)

Female (vs. Male)	0.68 (0.64-0.72)	0.70 (0.66-0.74)	0.89 (0.84-0.94)	0.86 (0.81-0.92)

Disease progression at baseline (CD4<200 and/or WHO Stage 3-4 vs. CD4 ≥ 200 and/or WHO Stage 1-2)	1.15 (1.07-1.23)	0.97 (0.90-1.04)	1.32 (1.24-1.42)	1.28 (1.20-1.38)

Post-ART initiation (vs. pre-ART)	1.34 (1.26-1.43)	1.28 (1.19-1.36)	0.91 (0.86-0.96)	1.50 (1.39-1.63)

Marital Status				
Single	1.00	1.00	1.00	1.00
Married	0.89 (0.83-0.96)	0.95 (0.88-1.03)	0.80 (0.75-0.86)	0.73 (0.67-0.79)
Other (widowed, divorced, separated)	0.74 (0.68-0.81)	0.86 (0.78-0.95)	0.86 (0.79-0.93)	0.78 (0.71-0.86)

Educational attainment				
None	1.00	1.00	1.00	1.00
Primary	1.10 (0.99-1.21)	1.06 (0.96-1.16)	0.85 (0.79-0.92)	0.79 (0.72-0.86)
Secondary/Tertiary	1.37 (1.26-1.49)	1.15 (1.06-1.26)	0.77 (0.72-0.82)	0.72 (0.66-0.78)

Enrollment year				
2008	1.00	1.00	1.00	1.00
2009	0.83 (0.77-0.88)	0.93 (0.86-0.99)	1.14 (1.06-1.22)	1.16 (1.07-1.25)
2010	0.61 (0.56-0.66)	0.75 (0.69-0.82)	1.29 (1.19-1.39)	1.27 (1.16-1.39)
2011	0.47 (0.42-0.52)	0.55 (0.49-0.62)	1.46 (1.34-1.59)	1.53 (1.38-1.70)
2012	0.22 (0.17-0.28)	0.25 (0.19-0.33)	1.49 (1.29-1.71)	1.50 (1.27-1.78)

* Adjusted models include all variables in the table
